# Psychiatric Illnesses, Somatic Complaints, and Treatments in a Tertiary Care Hospital in Khyber Pakhtunkhwa, Pakistan: A Cross-Sectional Study

**DOI:** 10.7759/cureus.43151

**Published:** 2023-08-08

**Authors:** Muhammad Noman K Wazir, Susan Kakakhel, Aqsa N Gul, Quratulain Awan, Almas F Khattak, Nowsher Yousaf, Fakhria Wahid

**Affiliations:** 1 Psychiatry, Northwest General Hospital and Research Center, Peshawar, PAK; 2 Physiology, Northwest School of Medicine, Peshawar, PAK; 3 Psychology, Islamia College, Peshawar, PAK; 4 Community Medicine and Research, Northwest School of Medicine, Peshawar, PAK; 5 Occupational Health Safety and Environment (OHS&E), Northwest General Hospital and Research Center, Peshawar, PAK

**Keywords:** psychiatry and mental health, cultural psychiatry, treatment options, somatic presentation of psychiatric illnesses, asia, pakistan, prevalence, khyber pakhtunkhwa, somatic symptoms, psychiatric illnesses

## Abstract

Background: Psychiatric disorders differ in frequency and symptoms based on the cultural and ethnic background of patients. This can make diagnosing and treating patients challenging globally. In Pakistan, most psychiatric patients report multiple somatic complaints. Our goal was to investigate the causes of these complaints, identify common psychiatric conditions, and analyze their various manifestations in clinical practice. We also aimed to identify ways to improve the quality of care provided to our patients.

Methodology: We collected and organized data by utilizing predetermined tables from a sample of 231 patients who visited the outpatient clinics. Inpatients were not included in this study because of the absence of a psychiatric unit at our facility. Patients’ past medical and psychiatric records were thoroughly examined, and pertinent information was extracted. The most common psychiatric disorders within the studied population were diagnosed based on the International Classification of Diseases, Tenth Revision (ICD-10) diagnostic criteria.

Results: In this study, a sample size of 231 was examined to determine the most common diseases (ICD-10) in males and females. In males, the most prevalent diseases were mixed anxiety and depression (MAD), depression, generalized anxiety disorder (GAD), bipolar affective disorder (BPAD), social phobia, and obsessive-compulsive disorder (OCD). Females, on the other hand, presented more with depression, GAD, mixed anxiety and depression, conversion or dissociative disorder, OCD, and panic attacks. Headaches were the most frequently reported symptom, experienced by 61.9% the of participants, followed by lethargy, extremity pains, palpitations, loss of appetite, heartburn or acidity, heaviness on the head, shoulder pains, bloating, dizziness, chest pains, hot flashes or shivering, and constipation. Meanwhile, a quarter of the males did not complain of any somatic symptoms, compared to 10% of the females. Additionally, 7.3% of females reported more than six somatic symptoms, compared to 5.7% of males. When it came to treatment preferences, 73.6% of the participants preferred medication over psychotherapy and over a combination of both.

The Statistical Product and Service Solutions (SPSS) Statistics version 22 (IBM SPSS Statistics, Armonk, NY, USA) was used to conduct a chi-square test of independence to analyze the obtained data. For post hoc analysis of quantitative data (i.e., the number of somatic symptoms reported by participants), one-way analysis of variance (ANOVA) was applied, followed by Tukey’s honestly significant difference (HSD) test.

Conclusions: This is the first comprehensive study of its kind for this population and region. It emphasizes that clinicians should be aware of the variety of somatic symptoms and psychiatric presentations among this population. Such awareness can improve clinical practices and reduce the burden on health services.

## Introduction

Patients often “psychologize” their feelings in the West, whereas psychological discomfort is typically “somatized” in non-Western communities [1]. Somatization was originally used in transcultural psychiatric literature by Arthur Kleinman (1977), who described it as “the expression of personal and social distress in an idiom of bodily complaints with medical help-seeking” [2].

Patients with anxiety and depressive disorders tend to report more physical symptoms than psychological ones in non-Western settings [3]. Although somatic symptoms including headaches, nebulous aches/pains, exhaustion, and dizziness frequently go hand in hand with psychiatric presentations, this physical presentation of mental diseases is not exclusive to non-Western countries [4].

These studies have shown that in several cultures, there exist discrepancies in the way physical symptoms are expressed. Individuals tend to manifest depression and other psychological issues primarily through somatic symptoms. This inclination toward somatic idioms, such as having a “headache,” “body ache,” or “heaviness on my head,” as opposed to “feeling low/feeling sad” to cope with emotional distress, can be attributed to the intense shame and stigma associated with mental illness in several Asian cultures. In such societies, somatic expression of psychological distress is viewed as a coping mechanism to garner social support from family and community and to find relief from daily responsibilities.

From a medical standpoint, somatization poses a distinctive difficulty for doctors because of the ambiguous nature of its diagnosis. Despite the exhaustive and expensive investigations that are conducted, physicians may still be reluctant to diagnose somatization because of the apprehension of missing an organic condition or wrongly categorizing the patient as a hypochondriac.

The province of Khyber Pakhtunkhwa (KP), previously known as Northwest Frontier Province (NWFP), is situated in the northwestern region of Pakistan, bordering Afghanistan. KP is the third-largest province in Pakistan in terms of both population and economy. As of the 2017 Census of Pakistan, the province has a population of approximately 35.5 million people, which accounts for 11.9% of Pakistan’s total population, with 52% males and 48% females. Moreover, around 1.5 million Afghan refugees also reside in the province. KP has been a significant battleground of militancy and terrorism since the 9/11 attacks in the USA in 2001.

The main motive behind this paper is to address the fact that most psychiatric patients seen in clinics in Pakistan seek treatment with a myriad of somatic complaints. The objective is not only to determine the prevalence of common psychiatric conditions and their distinct presentations in practice but also to identify opportunities to enhance the care provided to patients.

## Materials and methods

We collected data using convenience sampling from patients who visited the outpatient clinics at the Northwest General Hospital and Research Center, a 300-bed private hospital that serves the people of KP, Pakistan, and Afghanistan. Data were tabulated using predetermined tables by the interviewers. The International Classification of Diseases, Tenth Revision (ICD-10) criteria were used to identify and diagnose psychiatric illnesses. The study adhered to ethical standards set by relevant national and institutional committees on human experimentation and the Helsinki Declaration of 1975 revised in 2008. The Ethical Committee of the Northwest General Hospital and Research Center approved all procedures involving human subjects/patients, and verbal consent was obtained from all participants included in this cross-sectional study.

We also conducted a comprehensive literature review using multiple websites and written articles such as PubMed and the British Journal of Psychiatry (BJPsych). The articles included in the study focused on “somatic complaints in Asian or South Asian populations” and were chosen based on their relevance to the objectives and the population and to the refugees in Pakistan, the UK, and the USA.

The obtained data were analyzed using the chi-square test of independence with Statistical Product and Service Solutions (SPSS) Statistics version 22 (IBM SPSS Statistics, Armonk, NY, USA). The variables examined included gender, literacy status, and the presence or absence of somatic symptoms. Quantitative data, i.e., the number of somatic symptoms reported by the subjects, were analyzed using one-way analysis of variance (ANOVA). A post hoc analysis was conducted using Tukey’s honestly significant difference (HSD) test.

## Results

The total number of patients seen in the clinics was 231 (n=231), with a sample size consisting of 53% males and 47% females, and the mean age was 39.9 years. The majority of the patients seen belonged to the 18-65 years age group (84%) (<18 years: 20, 18-65 years: 195, and >65 years: 16). Age groups 20-29 and 50-59 were the most common among males and females, respectively (p<0.01). Patients who were educated or in school/college made up 43.2% of the study sample, whereas 56.7% of the subjects had no educational background. No significant relationship was found between education and gender.

**Figure 1:**
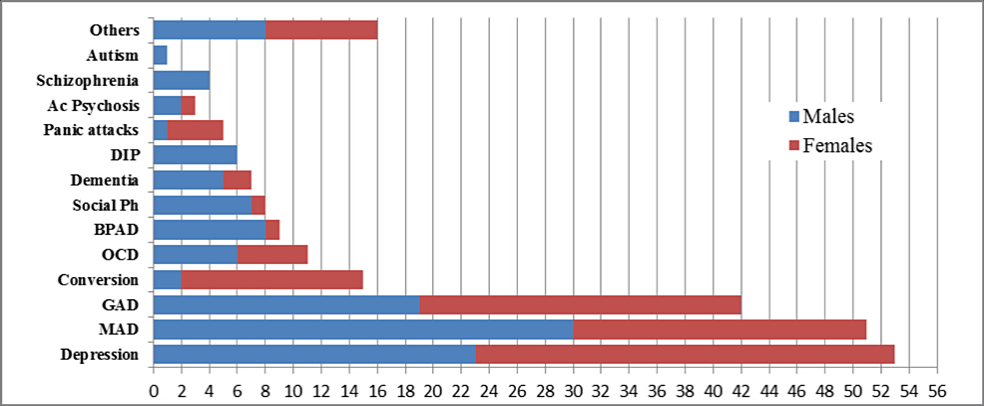
Prevalence of psychiatric illnesses by gender Ac Psychosis: acute psychosis, DIP: drug-induced psychosis, Social Ph: social phobia, BPAD: bipolar affective disorder, OCD: obsessive-compulsive disorder, GAD: generalized anxiety disorder, MAD: mixed anxiety and depression

The psychiatric disorders most commonly found in males were mixed anxiety and depression (MAD) (24.5%), depression (18.8%), generalized anxiety disorder (GAD) (15.5%), bipolar affective disorder (BPAD) (6.5%), social phobia (5.7%), obsessive-compulsive disorder (OCD) (5.7%), drug-induced psychosis (4.9%), dementia (4.09%), schizophrenia (3.2%), acute psychotic episode (1.6%), conversion or dissociative disorders (1.6%), autism (0.8%), panic attacks (0.8%), and others (6.5%). The most prevalent diseases in females were depression (27.5%), GAD (21.1%), MAD (19.2%), conversion or dissociative disorder (11.9%), OCD (4.5%), panic attacks (3.6%), dementia (1.8%), social phobia (0.9%), BPAD (0.9%), acute psychotic episode (0.9%), and others (7.3%) (Figure 1).

**Figure 2:**
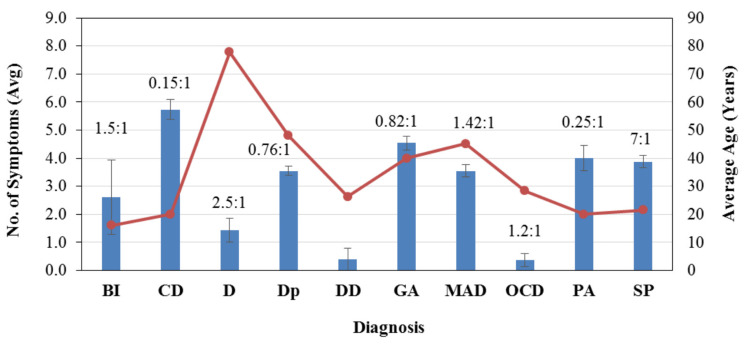
Number of somatic symptoms reported by patients diagnosed with different psychiatric conditions/disorders The bars and line represent the average number of symptoms reported by each of the groups ± standard error and average age, respectively. The male/female ratio is indicated by the data values above the bars. Note: Dual diagnoses prevailed in males only. BI: behavioral issues, CD: conversion/dissociative disorder, D: dementia, Dp: depression, DD: dual diagnosis, GA: generalized anxiety, MAD: mixed anxiety and depression, OCD: obsessive-compulsive disorder, PA: panic attacks, SP: social phobia

Conversion disorder was significantly associated with females compared to males; the opposite was true for BPAD (p<0.01). Conversion disorder and behavioral issues were more common in subjects under 20 years of age, whereas social phobia, depression, and dual diagnosis were significantly linked to patients in their 20s (p<0.0000001). The age-based distribution showed a strong association between dementia and patients aged 70-89 years. Moreover, patients with conversion disorder were significantly younger than those diagnosed with depression and mixed anxiety and depression (p>0.0000001) (Figure 2).

**Figure 3:**
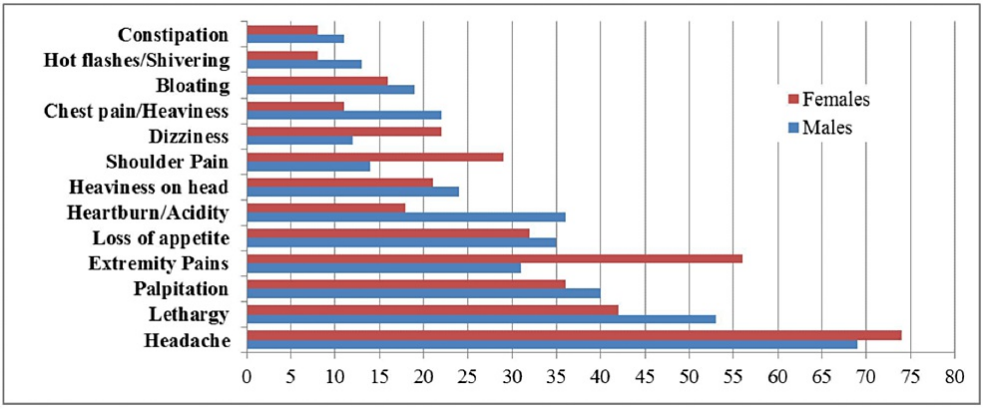
Most commonly reported somatic symptoms

Headaches, which were reported by 61.9% of the patients, were the most commonly experienced symptom, followed by lethargy (41.1%), extremity pains (37.6%), palpitations (32.9%), loss of appetite (29%), heartburn/acidity (23.3%), heaviness on the head (19.4%), upper neck/shoulder pain (18.6%), bloating (15.1%), dizziness (14.7%), chest pains (14.2%), hot flashes/shivering (9%) and constipation (8.2%) (Figure 3).

Frequently co-occurring symptoms included shivering and dizziness and shivering and chest tightness (p<0.0000001). Somatic symptoms were absent among 24.5% of males and 10% of females. Moreover, 44% of females reported fewer than three somatic symptoms, but 7.3% reported more than six symptoms. Among males, 41.8% reported between three to six somatic symptoms, while 5.7% reported more than six somatic symptoms. Overall, females reported more somatic symptoms than males at 89.9% for females and 75.4% for males. The absence of any somatic symptoms was significantly linked with males, whereas females were strongly associated with reporting six or more somatic symptoms (p<0.01). The age-specific distribution revealed that patients of age groups under 20 and 20-29 years reported a higher number of somatic symptoms (p<0.01).

Furthermore, patients diagnosed with OCD reported the least number of somatic symptoms (mean), while the highest was reported by patients with conversion disorder (p<0.0000001). The prevalence of extremity pain was significantly higher (p<0.0001) in females, followed by neck or shoulder pain (p<0.01), as compared with males. Heartburn or acidity was found to be more frequent among males (p<0.05). Furthermore, somatic symptoms such as shivering, constipation, chest tightness, and shoulder pain were associated with the age range of 20-29 years (p<0.01).

Treatment preference was also noted, such that 73.6% of the sample preferred medications over psychotherapy and over medications and psychotherapy combination (26.3%). Females preferred psychotherapy (alone or in combination with medications) at 34%, whereas males preferred medications alone as their treatment preference at 80.3%. Moreover, based on educational background, a strong link was established between literacy and psychotherapy as the preferable type of treatment. Conversely, illiterate patients chose medication over psychotherapy (p<0.000001).

Because of these multiple somatic complaints, it was not surprising that most patients we saw had also contacted numerous other physicians and specialists in the past. The majority (n=152 or 65.8%) had seen at least one psychiatrist or physician before presenting to us. Patients with their very first psychiatric presentation to our services were 62% compared to follow-up appointments of 38% (Table 1).

**Table 1:** Contacts made with different specialties/number of contacts made

Specialties	1	2	3	>4
Psychiatrists (n=61)	48%	25%	16%	11%
Physicians (n=33)	37%	21%	36%	6%
Cardiologists (n=10)	60%	40%	-	-
Gastroenterologists (n=12)	53%	21%	16%	10%
Neurologists/neurosurgeons(n=30)	50%	34%	8%	8%
Others (n=6)	67%	33%	-	-

## Discussion

Numerous studies have shown the importance of improving clinical care for people with mental health disorders, which is a developing concern. To gather information on health-related outcomes and their determinants, the World Health Organization (WHO) conducted the World Health Survey (WHS) on persons aged 18 and older. According to the study, compared to other chronic diseases, depression, either by itself or in comorbidity, produced the biggest decline in health. This emphasizes the necessity of making the treatment of depression and other psychiatric illnesses a top public health priority to lower disease burden and impairment and enhance population health [5].

Physical symptoms are commonly observed in depressed patients from all cultural groups. The percentage of patients in international clinics reporting unexplained physical symptoms ranges from 45% to 95%, with an average across all groups of 69% [6]. People from Asian cultures tend to experience negative, depression-like emotions amalgamated with somatic symptoms and interpersonal dissonance, signifying a tendency to present with multiple somatic distress complaints [7]. The prevalence of somatic symptoms is higher when compared to somatoform disorders and culturally bound somatic syndromes. This is because clients experiencing somatic symptoms are not required to meet specific diagnostic criteria [8]. However, research has also emphasized the importance and prevalence of somatic complaints as part of the distress experienced by patients in numerous cultures and ethnic populations [9,10].

Experiences of somatic symptoms and their prevalence, symptoms, and conceptualizations vary across cultures [11]. Understanding these somatic symptoms as part of psychological distress and offering support help reduce such symptoms and their severity [12]. Asian cultures place more emphasis on somatic complaints as a sign of distress, and this is because they believe the mind and body are one [13]. The way clients feel and articulate their suffering is influenced by the widespread stigma related to mental health [14]. According to research, physical discomfort experiences and medical diagnoses are more commonly accepted than psychological diseases [15]. Therefore, Asians are more likely to attend to a person’s physical needs than their psychological needs [16]. Excessive emotional outbursts, for example, are stigmatized by cultural norms as a sign of immaturity, weakness, or lack of self-control [13,16]. Clients who are repressing their emotions may start to feel pain in other areas of their bodies, which is more socially acceptable [10].

A strong therapeutic alliance can assist medical professionals in gathering more information and clarifying signs and diagnoses [13,14]. Somatization is more prevalent in those with lower levels of education or socioeconomic status and with a rural upbringing, as well as in ethnic groups that restrict the open expression of emotional suffering [17]. Effective tools to measure somatic symptoms might help clinicians provide appropriate treatment for such patients [18]. Clinicians who incorporate the somatic concerns of their Asian patients into their treatment strategies are more likely to achieve positive therapeutic results [19].

Some physicians focus solely on the biomedical model, which can lead to the neglect of psychosocial problems that often contribute to common psychiatric illnesses such as depression. This neglect manifests generally in the form of misdiagnosis, disregard of psychological distress or gratuitous investigations [20]. When patients with mental health issues are effectively treated, their somatic complaints and use of healthcare services decrease, indicating the need for an integrated biopsychosocial approach [21].

The demographic details, the prevalence of common psychiatric conditions, and somatic symptoms expressed in our study were as expected, with slight variations due to the population our hospital serves. More patients should have been included in the study, and expanding the data beyond private practices and hospitals would make studies like this more robust and viable.

Our observations showed that certain illnesses were more prevalent in males than in females, which can be explained by cultural variations and demographics. Females predominantly presented with depressive features, which can be attributed to their limited social interactions and lack of a strong social support network. Most were raised in shared family structures, which can sometimes be protective but can also have the opposite effect. It was noted that many married females had few interactions with their husbands because of their family structures, polygamy, or crowded living situations or the fact that their husbands were employed abroad and only came home occasionally.

Mental health care must be integrated as a core service in primary care in Pakistan, with support from specialist services. In addition to a cooperative network between stakeholders in the public, private, and non-governmental organizations to promote mental health care and advocate for changes in mental health policy, there is an urgent need for adequate training of general practitioners and for postgraduate training of mental health professionals [22].

Studies indicate that being exposed to traumatic events is linked to a higher prevalence of severe mental health issues in Pakistan, where these issues have alarmingly increased in recent decades [23,24], linked to both the country’s ongoing violence [25,26] and the disruption of its social order [27].

Factors positively associated with anxiety and depressive disorders include the female sex, middle age, low-level education, financial difficulty, filling the role of a housewife, and relationship problems. The mean overall prevalence of anxiety and depressive disorders in the community population was 34% (ranging from 29% to 66% for females and from 10% to 33% for males) [28]. Women in Pakistan often face domestic violence and restrictions on equal rights, particularly in rural settings. Other risk factors are being divorced or widowed, conflict with in-laws, financial strain, and the status of being a housewife rather than being employed [29].

Because of a lack of education, a poor grasp of psychiatry and psychological problems, budgetary constraints, the impossibility of providing counseling services, and other factors, only 25% of the patients seen in our study were willing to participate in any kind of psychological intervention.

The use of standardized questionnaires, such as the Patient Health Questionnaire-15 or the Somatic Symptom Scale 8, could provide insightful information about the patient’s mental state and the need for a referral to/or treatment from a trained professional. Clinicians need to restrict the use of needless tests and treatments, consult and work more closely with mental health specialists, and demonstrate more empathy with an emphasis on the body-mind connection.

The study has some limitations; it only included patients presenting to a private hospital in tertiary care settings and did not include any admitted patients because of the lack of a dedicated inpatient unit. To make such studies more robust, including a greater number of participants from inpatient, outpatient, and community settings over a longer period would be more beneficial.

## Conclusions

To the best of our knowledge, this is the first comprehensive study of its kind catering to this specific part of the world and a sample from the population. This study emphasizes the need for clinicians to recognize somatic symptoms and different presentations of psychiatric illnesses in their clinical practices.

By using standardized questionnaires and limiting excessive investigations or unnecessary referrals, clinicians can recognize these symptoms as part of a psychiatric illness and provide better quality care to patients. Psychoeducation of patients, their relatives, society as a whole, and healthcare professionals is necessary and can be achieved through the more active involvement of the government and private or public sector by organizing walks, dialogues, and programs, using print and social media, and establishing centers in the community where mental illnesses can be adequately screened and treated.

These steps can produce a mental health literate society, reduce stigmatization, and ensure appropriate care is provided to the population’s mental health needs.
